# 

**Published:** 1886-03

**Authors:** 


Page.

ORIGINAL COMMUNICATIONS:

Intubing the Larynx for Acute Catarrhal Laryngitis. Albert B. Strong ........ 193

A Complicated Labour. John D. Skeer ........ 199

A Case of Acute Albuminuria. C. H. H. Hall ........ 201

Quinsy as a Rheumatism. J. Suydam Knox ........ 208

Intubation of the Larynx, with History of Cases. F. E. Waxham ........ 214

EDITORIAL:

The Cook County Insane Asylum ........ 233

DOMESTIC CORRESPONDENCE:

Chloral Hydrate in the Treatment of the Hyperemesis of Pregnancy. D. D. Marr ........ 238

SOCIETY REPORTS:

*Philadelphia Academy of Surgery.* Notes on the new Antiseptics, Hydronaphthol and the Potassio-Mercuric Iodide. R. J. Levis ........ 239

A Pin for Treatment of Fracture of the Patella. T. G. Morton ........ 247

A Case of Obstructive Jaundice—Cholecystotomy for Impacted Gall-Stones. W. W. Keen ........ 248

Deformity of the Upper Extremities. W. W. Keen ........ 255

*Chicago Medical Society, Meeting of January 18th.* Mutual Protection Against Blackmail. E. J. Doering. 257

Determination of Sex in Utero. H. D. Valin ........ 265

*The Chicago Gynæcological Society, Meeting January 15th.* Watery Discharges of Pregnant Women. C. W. Earle ........ 268

Report of a Case of Hydatiform Pregnancy. E. J. Doering ........ 268

Intubation of the Larynx. F. E. Waxham ........ 268

BOOK REVIEWS:

Facts and Mysteries of Spiritism. Joseph Hartman ........ 287

ITEMS ........ 288

